# Proline: A Reliable Biochemical Marker of Plant Abiotic Stress Tolerance?

**DOI:** 10.3390/plants15162478

**Published:** 2026-08-15

**Authors:** Delia Maria Luca, Marius-Nicuşor Grigore, Oscar Vicente

**Affiliations:** 1 Faculty of Biology, “Alexandru Ioan Cuza” University, 20A Carol I Bd, 700506 Iasi, Romania; delia.luca@student.uaic.ro; 2 Doctoral School of Biology, “Alexandru Ioan Cuza” University, 20A Carol I Bd, 700506 Iasi, Romania; nicusor.grigore@uaic.ro; 3 Institute for the Conservation and Improvement of Valencian Agrodiversity (COMAV), Universitat Politècnica de València, Camino de Vera s/n, 46022 Valencia, Spain

**Keywords:** climate change, drought, salinity, abiotic stress responses, stress tolerance, osmolytes

## Abstract

Climate change is placing global agriculture under growing pressure, as plants must withstand extreme environmental conditions such as drought and high salinity, both inducing osmotic and oxidative stress. As part of their survival strategies, plants accumulate protective molecules (osmolytes), including the amino acid proline. For decades, plant biology has largely assumed that high proline accumulation under stress signals strong stress tolerance. However, this review challenges that “proline-centric” perspective. Analyses across a wide range of plant species reveal a more complex picture. Stress-induced proline accumulation is not universal: in some species, proline levels remain relatively unchanged, with other metabolites acting as functional osmolytes, or increase only in response to artificially applied severe stress conditions. Even when proline increases, its absolute concentrations may be too low to contribute significantly to osmotic adjustment. Nevertheless, proline may still be involved in stress tolerance mechanisms through its additional roles, detoxifying reactive oxygen species (ROS), directly stabilising proteins or acting as a stress signalling molecule. Comparative analyses of genetically related taxa with varying degrees of stress tolerance sometimes show negative correlations between proline accumulation and tolerance, with higher proline concentrations measured in the most sensitive genotypes. Overall, the evidence indicates that proline‘s role in plant survival is highly context-dependent and strongly influenced by genetic background and must therefore be evaluated on a case-by-case basis. Distinguishing whether proline acts as an adaptive defence or merely as a biochemical marker of physiological strain under stress is essential for accurately assessing plant stress tolerance.

## 1. Introduction

It is well established that climate change intensifies the impact of environmental factors generating abiotic stress in plants—such as salinity, drought, heatwaves, or heavy rainfall and flooding—to the detriment of crop yields, food security, and the biodiversity of wild species [1,2]. To survive these effects, plants have evolved a complex, multi-layered defence strategy. This strategy involves the tight regulation of ion transport and homeostasis, particularly through mechanisms that restrict the entry of sodium (Na^+^) and chloride (Cl^−^) ions into the transpiration stream or sequester them safely within the vacuole to prevent cytoplasmic toxicity [3]. At the same time, since different abiotic stresses generate oxidative stress as a secondary effect, plants activate a wide array of antioxidant systems, which include both enzymatic components—for example, superoxide dismutase (SOD), catalase (CAT), ascorbate peroxidase (APX) or glutathione reductase (GR)—and antioxidant metabolites, such as glutathione, ascorbate, and phenolic compounds [4,5]. The coordinated action of these antioxidant defences helps maintain cellular redox balance by scavenging the reactive oxygen species (ROS) generated in plants affected by environmental stress factors [6,7].

However, a plant’s ability to maintain water uptake and limit cellular dehydration under hyperosmotic conditions largely depends on osmotic adjustment. This process involves the active synthesis and intracellular accumulation of low-molecular-weight organic compounds known as compatible solutes or osmolytes. These compounds form a chemically diverse group, including quaternary ammonium compounds, sugar alcohols, soluble sugars and amino acids [8,9].

Amongst these compatible solutes, the amino acid proline (Pro) occupies a distinctive—and often controversial—position in plant stress physiology. Pro accumulation is one of the most widely reported metabolic responses to abiotic stress and has been documented across a broad range of plants, both crops and wild species, including, for example, herbaceous crops [10,11], cereals [12,13], ornamentals [14,15], halophytes [16,17,18] or forest trees [19,20,21]. The role of Pro in mediating plant responses to abiotic stress, particularly drought and salinity, has therefore been a cornerstone of plant physiological research for decades, and it is widely accepted that Pro contributes to stress tolerance mechanisms. Nevertheless, a critical examination of the literature reveals considerable ambiguity and, in some cases, contradictory interpretations. Many studies infer that Pro acts as a primary osmolyte solely on the basis of its increase under stress, often without considering that the amount of proline accumulated, while statistically significant compared to the non-stressed control, may not be physiologically significant for osmotic adjustment. The assumption that Pro is directly involved in the mechanisms of stress tolerance in plants is so widely accepted that in many publications Pro is the only potential osmolyte quantified, disregarding the possible contribution of additional compatible solutes, such as soluble sugars, sugar alcohols or glycine betaine. The additional roles of Pro in the responses of plants to abiotic stress, as an “osmoprotectant” or low-molecular-weight chaperone, directly stabilising proteins and other macromolecules under stress conditions, as an ROS scavenger, or as a stress signalling molecule [22,23,24] make the assessment of its contribution to stress tolerance even more difficult.

**Figure 1:**
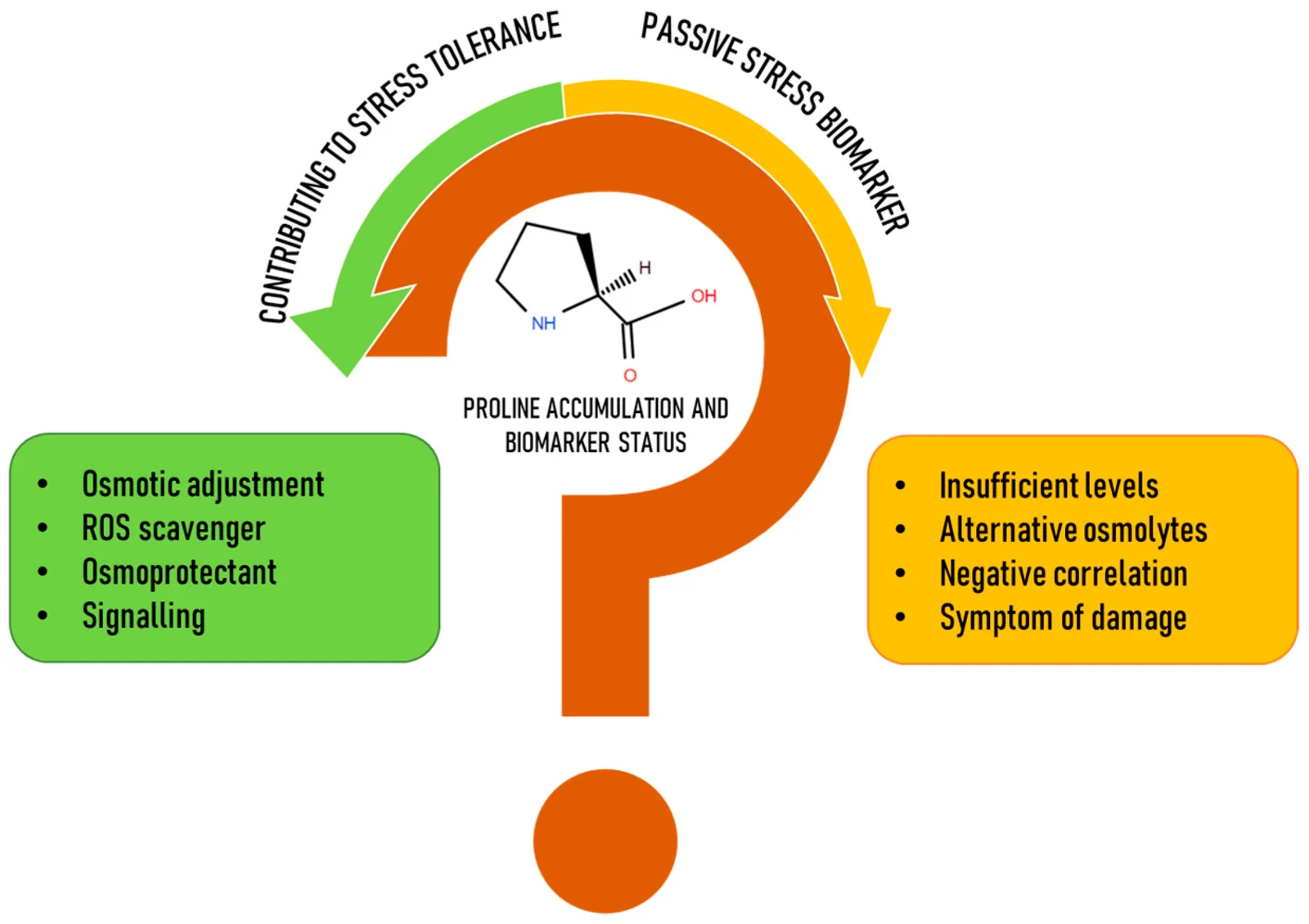
Proline: Contributing to stress tolerance versus passive stress biomarker.

Notwithstanding the relevance of other stress responses, such as the control of ion transport or the activation of antioxidant systems, in the mechanisms of tolerance, this review focuses specifically on the role of Pro. We attempt to critically evaluate the functional significance of Pro accumulation across diverse plant taxa to address persistent uncertainties regarding its role in stress tolerance. Based on published data, including work from of our group during the last decade, we challenge the prevailing “proline-centric” paradigm by examining key factors frequently overlooked in the literature: the functional specificity of Pro compared with alternative osmolytes; the physiological relevance of absolute concentration thresholds required for osmotic adjustment; the *ecological* relevance of Pro accumulation in those cases where Pro biosynthesis is triggered only under—artificially applied—extreme stress conditions, which the plants would not normally encounter in their natural habitats; or the observation, when comparing several related taxa, of negative correlations between the stress-induced Pro accumulation and their relative degree of tolerance. By synthesising available evidence, this review proposes a more nuanced framework for distinguishing Pro accumulation as a passive biomarker of stress-induced cellular damage from its role as a functional adaptive stress-tolerance mechanism (Figure 1).

After a general overview of Pro metabolism and its regulation, we highlight the challenges and limitations in determining the role of Pro in the mechanisms of responses to abiotic stress. Then, we provide examples from the literature, either supporting Pro’s function as an active osmolyte or, rather, as a passive stress biomarker. We focus primarily on the most common studies, those showing the accumulation of Pro in response to drought and high salinity or, in some cases, to other abiotic stressors such as high temperature, hypoxia or heavy metals, and its effects on osmotic adjustment and stress tolerance. Most of these studies are limited to the measurement of Pro concentrations in plant extracts, although some publications also include mechanistic aspects; for example, the analysis of the expression of genes involved in Pro biosynthesis and degradation.

Other experimental approaches, such as the modification of endogenous Pro concentrations in transgenic plants or the exogenous application of the amino acid to stressed plants, have also provided information supporting its functional role in stress tolerance mechanisms and have been discussed in several reviews (e.g., [22,25,26]). These data, however, are not included in this article, mostly centred on the endogenous biosynthesis and accumulation of Pro in response to abiotic stress treatments. The additional functions of Pro in plant metabolism, growth, and development that are not directly related to stress response mechanisms (e.g., [23,24,27]) are also outside the scope of this review.

## 2. Proline Metabolism

As mentioned above, Pro levels usually rise in response to various stresses, owing to its properties as a compatible solute and osmoprotectant [28]. Pro accumulation depends on its biosynthesis, breakdown, transport, and utilisation, all of which are closely regulated by the developmental stage, environmental challenges, and genetic mechanisms [22]. The relationship between Pro levels and stress resilience has shown variability across species, genotypes, and stress conditions.

### 2.1. Biosynthetic Pathways

**Figure 2:**
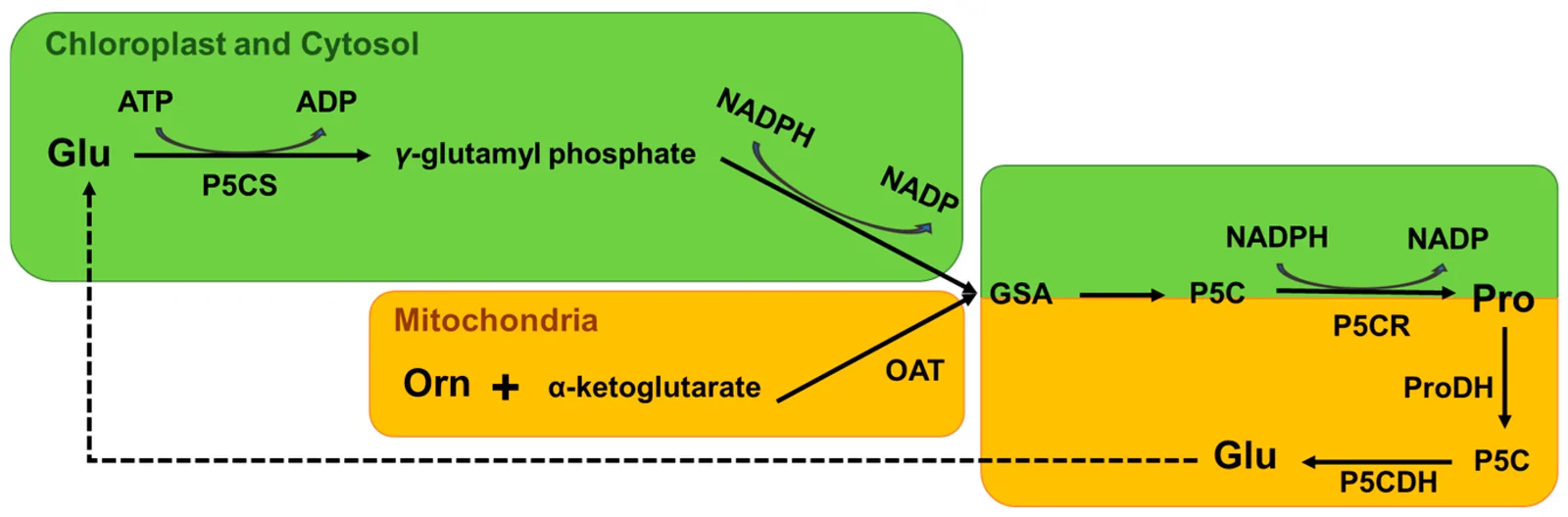
Metabolic pathways of proline biosynthesis and catalabolism. Glu: glutamate; Orn: ornithine; GSA: glutamate-γ-semialdehyde; P5C: pyrroline-5-carboxylate; P5CS: pyrroline-5-carboxylate synthetase; OAT: ornithine-δ-aminotransferase; P5CR: pyrroline-5-carboxylate reductase; ProDH: proline dehydrogenase; P5CDH: pyrroline-5-carboxylate dehydrogenase.

Proline synthesis occurs in both the cytosol and chloroplasts, with the isoforms of pyrroline-5-carboxylate synthetase (P5CS), the first enzyme in the pathway, showing differential localisation: P5CS1 is predominantly cytosolic and stress-inducible, whereas P5CS2 is constitutively expressed in both compartments, supporting developmental Pro requirements [7]. Glutamate serves as the primary precursor in this metabolic route, where P5CS catalyses the ATP-dependent phosphorylation of glutamate to γ-glutamyl phosphate, followed by its NADPH-dependent reduction to glutamate-γ-semialdehyde (GSA), which spontaneously cyclises to form pyrroline-5-carboxylate (P5C). Subsequently, P5C reductase (P5CR) completes the process by reducing P5C to proline [8,29] (Figure 2). Alternatively, ornithine (Orn) can act as a precursor for Pro biosynthesis. In this secondary mitochondrial pathway, ornithine-δ-aminotransferase (OAT) catalyses the transamination of ornithine with α-ketoglutarate to produce glutamate-γ-semialdehyde (GSA), which then follows the previous pathway to form P5C and Pro [8]. This specific biosynthetic route becomes particularly active when plants experience nitrogen limitation and during seedling development [30,31] (Figure 2).

### 2.2. Catabolic Pathways

Proline degradation takes place exclusively within the mitochondria and relies on the successive action of proline dehydrogenase (ProDH) and pyrroline-5-carboxylate dehydrogenase (P5CDH). ProDH catalyses the first step of Pro catabolism and is a key regulatory enzyme whose expression is tightly controlled in response to developmental and environmental signals [32,33,34]. P5CDH, also essential for Pro catabolism, may function as a protein complex with ProDH in mitochondria, and the coordinated regulation of both enzymes determines the flux through the catabolic pathway [34,35,36]. Together, their activity generates reducing power that actively contributes to ATP synthesis through oxidative phosphorylation. ProDH is a FAD-containing enzyme anchored to the inner mitochondrial membrane, where it transfers electrons from Pro oxidation directly to the ubiquinone pool of the respiratory electron transport chain, contributing to the proton-motive force and ATP synthesis [23,25,32]. Recent evidence suggests ProDH may function in a protein complex with P5CDH and OAT, potentially facilitating coordinated substrate channelling and electron transfer [33].

Ultimately, this catabolic pathway converts Pro to glutamate via ProDH and P5CDH (Figure 2). Glutamate can then be further metabolised by glutamate dehydrogenase, in a separate process, to 2-oxoglutarate (OG), a vital intermediate of the tricarboxylic acid (TCA) cycle, thereby yielding additional reducing equivalents to sustain cellular energy metabolism [37].

### 2.3. Regulatory Mechanisms of Pro Biosynthesis and Catabolism

The regulation of Pro metabolism extends beyond hormonal control, as it is governed by a complex network of signalling cascades and molecular mechanisms. A variety of triggers can induce the increase in endogenous Pro concentrations, ranging from reactive oxygen species like hydrogen peroxide (H_2_O_2_) to abscisic acid (ABA)-dependent and independent pathways, as well as calcium signalling and MAP kinase cascades [7]. Under salt stress, for instance, calcium-dependent signalling—involving phospholipase C, calcium-dependent protein kinases (CDPKs), and calmodulin-binding transcription activators (CaMTAs)—plays a particularly pivotal role in driving Pro accumulation [38,39]. Meanwhile, MAP kinase modules help bridge the gap between stress perception and metabolic output by modulating the expression of key biosynthesis genes such as *P5CS1* [40].

Focusing on the molecular machinery, Pro homeostasis is fine-tuned by an array of transcription factor families—including bZIP, MYB, Homeobox, CCAAT-binding, DREB, and NAC proteins—alongside epigenetic regulators such as the histone methylase CAU1 and the chromatin-remodelling complex FRI. Adding another layer of complexity, the cell also controls Pro levels through alternative splicing of the P5CS1 transcript and post-translational modulation of P5CR activity [23].

Phytohormones are also key coordinators in this process. Jasmonic acid (JA), for example, enhances Pro accumulation by turning on its biosynthetic genes, whereas ABA directly stimulates the transcription of those genes [28]. These signals feed into a broader transcriptional network that balances Pro biosynthesis and degradation, allowing the plants to adjust their Pro pools dynamically in response to changes in the environmental conditions.

The expression of Pro-related genes is also highly responsive to specific stresses. For example, in rice plants under hypoxia, Pro content increases through the simultaneous activation of *OsP5CS* genes and the repression of the catabolic gene *OsProDH* [41]. Similar regulatory mechanisms have been observed during temperature stress. In cold-stored cucumber fruit, *CsP5CS* expression and P5CS activity rise, boosting Pro biosynthesis. This effect intensifies in the presence of melatonin, which further enhances P5CS and OAT activities while suppressing both *CsPDH* expression and PDH activity, ultimately driving Pro levels even higher [42].

Therefore, Pro metabolism is regulated by complex hormonal and metabolic networks involving ABA, ethylene, JA, reactive oxygen species, calcium signalling, and multiple transcription factors. Consequently, Pro levels can vary in response to different external and endogenous signals and in turn affect different metabolic processes, complicating the assessment of its specific functions in stress tolerance mechanisms.

## 3. Assessment of Pro Function(s) in Abiotic Stress Responses: Challenges and Limitations

As mentioned above, Pro accumulation is one of the most widely documented responses of plants to abiotic stress. It has generally been assumed that its primary physiological role is to contribute to osmotic adjustment under conditions that cause cellular dehydration, such as drought, salinity, extreme temperatures, or heavy metal contamination in the soil. However, it is at present recognised that, apart from acting as a compatible osmolyte, Pro additional functions—as a reactive oxygen species (ROS) scavenger, molecular chaperone, stabiliser of proteins and membranes, redox buffer, and signalling molecule—may be essential in the mechanisms of plant responses to abiotic stress [22,25,43]. Despite the extensive literature, however, establishing the actual physiological contribution of Pro to stress tolerance and the relative relevance of its varied proposed roles remains remarkably difficult. Several methodological and conceptual limitations complicate the interpretation of experimental results and the comparative analysis of independent experiments.

Probably, the most important limitation is that Pro is practically always quantified in whole-tissue extracts, often using spectrophotometric methods such as the classic acid ninhydrin assay [44]. These assays provide average tissue concentrations but cannot determine Pro concentrations in different subcellular compartments, which represent the physiologically relevant values. This limitation is particularly important when evaluating Pro’s osmotic role because cytosolic, rather than total cellular, concentrations determine osmotic adjustment. To our knowledge, there is a single publication describing the direct quantification of Pro in intracellular compartments of intact leaves [45] and a few studies showing the possibility of determining the subcellular concentration of different metabolites after fractionation in non-aqueous gradients, although no specific Pro measurements were reported [46,47].

Comparisons of different published studies are further complicated by the lack of standardised Pro concentration units. Proline can be expressed in terms of sample fresh weight (FW) or dry weight (DW), or relative to protein or chlorophyll contents, making quantitative comparisons extremely difficult. As abiotic stress generally causes plant dehydration, the observed differences in Pro contents between stressed and control plants, when expressed in FW terms, can be partly due to differential water loss. Similarly, protein and chlorophyll contents are not appropriate for normalisation, as their concentrations are often altered under stress. Expressing Pro concentrations in relation to the sample dry weight, for example, as µmol g^−1^ DW, appears to be the most convenient choice. Unfortunately, it is generally not possible to recalculate Pro contents from data provided in publications that use other units.

The physiological significance of the measured Pro concentrations also remains controversial. Even though stress often induces statistically significant, many-fold increases in Pro levels, the estimated cellular concentrations are frequently too low—in the low millimolar range—to contribute to osmotic adjustment. Even when Pro accumulates to levels high enough to exert substantial osmotic effects, it is generally unclear how significant its contribution is to stress tolerance mechanisms, compared with other organic osmolytes and inorganic ions, partly because many studies quantify only Pro, ignoring other major compatible solutes. Indeed, only a few publications report attempts to calculate the *relative* contributions of Pro, additional organic osmolytes, and inorganic ions to total osmotic potential (e.g., [48,49,50]). Interestingly, these studies generally find that inorganic ions dominate total osmotic adjustment, whereas Pro, or organic osmolytes in general, play a comparatively modest osmotic role, even when their contents increase dramatically in response to the stress treatment (reviewed, for example, in [51,52].

Nevertheless, even without a major quantitative effect on whole-cell osmotic potential, Pro may still contribute significantly to osmotic adjustment under stress if it reaches high enough concentrations in the cytoplasm. According to the widely accepted “ion compartmentalisation hypothesis”, initially developed from studies on salt-tolerant species, plants preferentially sequester Na^+^ and Cl^−^ ions in the vacuole, where they provide most of the osmotic potential while avoiding their toxic effects in the cytoplasm. To balance the osmotic pressure generated by vacuolar ions, “compatible solutes”, including Pro, which do not interfere with normal cellular metabolism, accumulate in the cytoplasm [53,54]. In the absence of direct quantification of ion concentrations in the vacuole and of organic osmolytes in the cytoplasm, Pro content can be roughly estimated from average values of tissue water content and the relative volume of the cytoplasm in relation to the whole cell volume. Let us consider, for example, that the Pro content in a sample of typical adult leaves has been determined to be 100 µmol g^−1^ DW. The average water content of adult leaves is 80% of the fresh weight, the vacuole represents about 80% of the whole cell volume, and the cytoplasm about 15% [55]; therefore, Pro concentration will be ca. 25 mM in terms of tissue water content and >150 mM in the cytoplasm, assuming that Pro accumulates exclusively in this subcellular compartment. Obviously, these calculations are very approximate, as they depend on the specific tissue type; for example, the water content and vacuole volume in succulent leaves will both exceed 90%. Also, probably Pro is not completely excluded from the vacuole. In any case, accumulation to concentrations around and above 100 µmol g^−1^ DW appears to be an appropriate theoretical threshold for assigning Pro a relevant role in cellular osmotic adjustment, with decreasing contribution at lower concentrations.

## 4. Proline as a *bona fide* Functional Osmolyte

In this section, we will describe and briefly discuss some of the results reported in hundreds of publications that, according to the threshold suggested above, would clearly support Pro participation in cellular osmotic adjustment, providing the basis for the generally accepted view of this amino acid as a *bona fide* functional osmolyte.

In some wild taxa adapted to natural stressful environments, Pro accumulates to relatively high concentrations in response to severe stress treatments. For example, in four Mediterranean *Limonium* species, *L. virgatum*, *L. girardianum*, *L. narbonense* and *L. santapolense*, Pro and fructose were identified as the major functional osmolytes under salt stress, playing a more significant role than other compatible solutes such as glycine betaine (GB) or sucrose [56]. Upon exposure to 800 mM NaCl, Pro accumulation increased significantly but unevenly across all species, with *L. virgatum* showing the most pronounced response, reaching nearly 470 μmol g^−1^ DW (a 25-fold increase over the non-stressed control), followed by *L. girardianum* and *L. narbonense*. On the other hand, in *L. santapolense*—a narrow endemism from the south of the Alicante province, in the Spanish Mediterranean coast—Pro increased only 5-fold, up to about 100 μmol g^−1^ DW, a concentration still high enough to contribute significantly to osmotic balance, although not so much as fructose, which accumulated up to nearly 1 mmol g^−1^ DW in *L. santapolense* and *L. narbonense*, about double the concentration than in the other two species [56].

Plants of the same four species were also subjected to water deficit stress under controlled conditions, and all showed to be relatively resistant to drought. This tolerance is partly based on constitutive mechanisms such as the active transport to the shoots of mono- and divalent ions (Na^+^, Cl^−^, K^+^, Mg^2+^, Ca^2+^), which showed significantly higher concentrations in leaves than in roots, even in non-stressed control plants, in agreement with the critical role of inorganic ions in cellular osmotic adjustment. Interestingly, contrary to their relative responses to salt stress, *L. santapolense* was the species that showed the most strongly induced responses to the water stress treatment, including a much higher relative increase in leaf Pro contents, reaching ca. 120 µmol g^−1^ DW, compared to the other three species. Higher accumulation of fructose and the stronger activation of antioxidant enzymes (particularly superoxide dismutase and ascorbate peroxidase) were also observed for this species. This differential behaviour of *L. santapolense*, confirmed by Principal Component and cluster analyses, agrees with the fact that it grows in a more arid environment than the natural habitats of the other three taxa [57].

**Figure 3:**
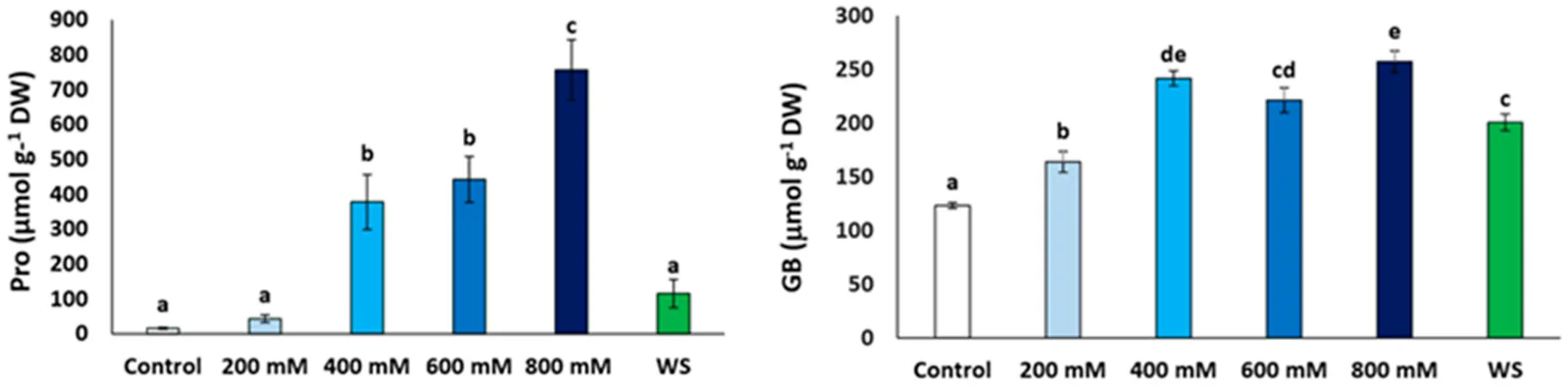
Proline (Pro) (**left** panel) and glycine betaine (GB) (**right** panel) contents in leaves of *Limonium augustebracteatum* plants after one month of treatment with the indicated NaCl concentrations or one month of water stress (WS, complete withholding of irrigation). Means ± SE, *n* = 6. In each panel, different lowercase letters above the bars indicate significant differences between treatments, according to Tukey’s test (α = 0.05). Adapted from [17].

In another *Limonium* species, *L. augustebracteatum* (Figure 3), Pro accumulation increased linearly by up to 48-fold (~760 µmol g^−1^ DW) in the presence of 800 mM NaCl. Notably, this response was absent under water stress conditions, suggesting that, for some species, Pro biosynthesis and accumulation can serve as a specialised adaptive mechanism for salinity tolerance rather than being a generic response to dehydration. On the other hand, glycine betaine also accumulated to high concentrations in *L. augustebracteatum*, up to 250 µmol g^−1^ DW, in this case in response to both increased salinity and a water deficit treatment [17]. It should be noted that GB concentration is relatively high in the non-stressed control, suggesting a constitutive mechanism of defence against stress, which is characteristic of GB-accumulating taxa (see below data for *Salicornia* species). Moreover, the simultaneous accumulation of both osmolytes to similar levels is inconsistent with the notion proposed by some authors that species that use Pro as the major functional osmolyte are poor GB accumulators, and vice versa (e.g., [58]), which holds true only for some species or genera. In fact, *Limonium* species are a good example of plants that use a diverse array of compatible solutes in their responses to stress [17,56,59].

Contrary to the previous example, in the halophyte *Sesuvium portulacastrum,* severe water deficit (25% field capacity) induced a progressive Pro accumulation, reaching ~300 µmol g^−1^ DW after one month of treatment. Interestingly, the concentration of mineral ions (Na^+^, K^+^, Cl^−^) also increased in leaves of water-stressed plants, supporting once again the essential contribution of inorganic ions to osmoregulation, in this case alongside Pro. [60].

**Figure 4:**
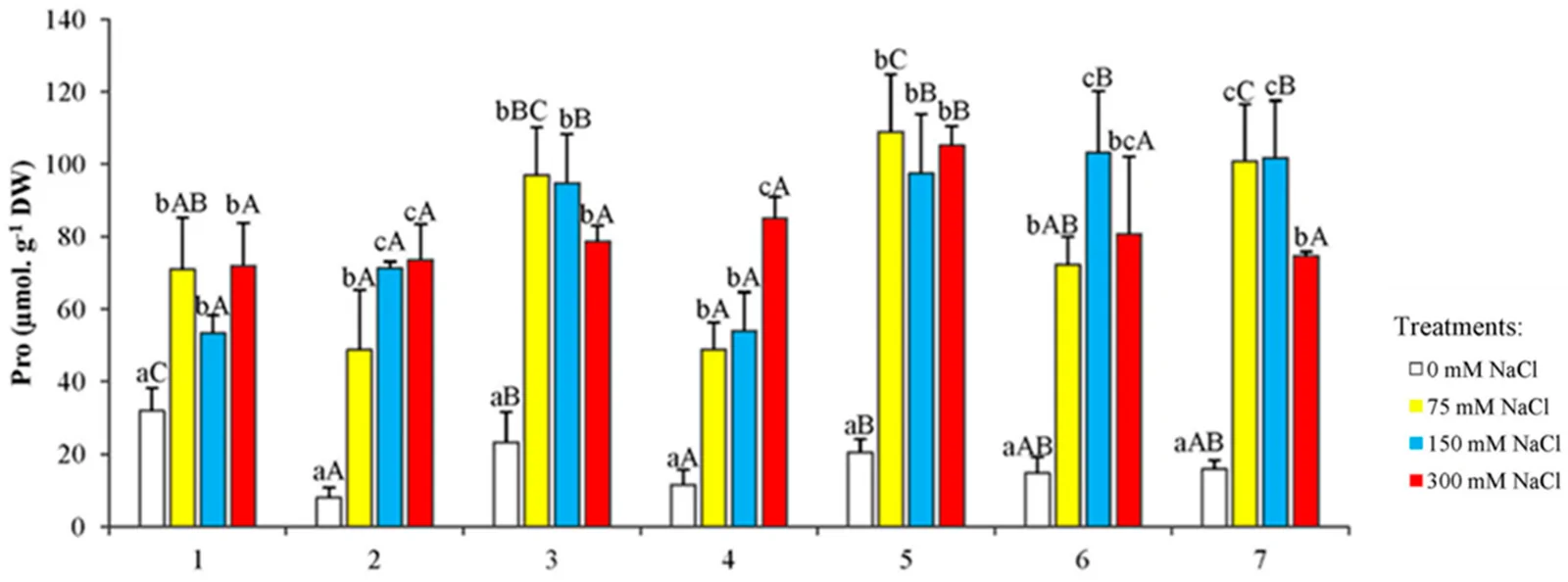
Variation of proline (Pro) content in seedlings from seven Romanian Carpathian populations of *Picea abies*, after 6-week treatments with the indicated NaCl concentrations. Data are means ± SD (*n* = 3). For each population, different lowercase letters above the bars indicate significant differences between treatments, and capital letters indicate significant differences between populations undergoing the same treatment, according to the Tukey test (α = 0.05). Adapted from [19].

Proline accumulation in response to salt stress is not limited to salt-tolerant taxa such as the halophytes mentioned above. In seedlings from seven *Picea abies* (Norway spruce) populations from the Romanian Carpathian Mountains, salt stress triggered a general and significant increase in needle Pro concentrations. The magnitude of this response varied quantitatively amongst populations, but the maximum Pro levels were roughly similar, ranging from 80 to 120 μmol g^−1^ DW under severe stress (300 mM NaCl) (Figure 4). No differences in salt tolerance were observed amongst the seven provenances, which was expected since the habitats where the trees grow do not include saline environments that could have induced the selection of more salt-tolerant genotypes. The positive correlation between Pro accumulation and salinity intensity was confirmed by Principal Component Analysis, supporting the role of Pro as a general stress marker. However, the authors noted that this correlation was statistically less significant than that observed for other measured variables, such as total phenolic compounds or sodium (Na^+^) and potassium (K^+^) contents, which should be considered as more reliable biomarkers of salt stress in this species [19].

The responses of the *P. abies* populations to severe water deficit—seedlings maintained for 42 days without irrigation—differed from those to salinity stress. Needle Pro contents in stressed seedlings varied widely amongst populations and were lower than those measured under severe salt stress, below 70 μmol g^−1^ DW in all cases. Two populations did not show any significant increase in Pro contents in response to the water stress treatment; those were the most drought-tolerant, according to other measured parameters and in agreement with the climatic characteristics of their geographical locations. Conversely, the most sensitive population exhibited the highest relative increase, ca. 8-fold, reaching concentrations of about 60 μmol g^−1^ DW. Therefore, in *P. abies*, Pro does not seem to play any relevant role in the mechanisms of drought tolerance but rather acts as an indicator of the level of stress affecting the plants [20].

Plants can fine-tune Pro biosynthesis and accumulation depending on the type and severity of the applied stress treatment. For example, *Portulaca oleracea* relied solely on stomatal regulation to respond to moderate salinity (100 mM NaCl), maintaining stable Pro and malondialdehyde levels comparable to those of the non-stressed controls. However, exposure to severe salinity (300 mM NaCl) triggered a distinct shift in strategy: stomatal conductance decreased early on, followed by a transition from C4 photosynthesis to Crassulacean Acid Metabolism (CAM) to preserve carbon fixation. Crucially, this high-salinity level induced a large (ca. 11-fold) increase in leaf Pro contents, reaching 32.12 µmol g^−1^ FW by day 22 [61]; this concentration easily exceeds 300 µmol g^−1^ in terms of dry weight, considering the highly hydrated, succulent nature of this species’ leaves. At these high concentrations, Pro can efficiently drive water uptake, supporting the authors’ conclusions regarding its primary functional role in osmotic adaptation to high salinity [61].

This trend is highly responsive to biological and chemical interventions. In pepper (*Capsicum annuum*) seedlings, Pro levels increased in response to eight-day treatments under different stress conditions (drought, salinity, heavy metals) [62]. Interestingly, inoculation with plant growth-promoting *Bacillus amyloliquefaciens* bacteria under salt stress further enhanced Pro accumulation to a peak of 9.74 mg g^−1^ DW (approximately 85 µmol g^−1^ DW). Although at this concentration, Pro’s contribution to osmotic adjustment may not be highly relevant, under these conditions the plants maintained high chlorophyll and sugar levels while effectively reducing oxidative stress markers like hydrogen peroxide [62]. Similarly, in chickpea plants, chilling stress causes endogenous Pro to spike dramatically, up to 230 μmol g^−1^ DW, a concentration that increased even more, to 310 μmol g^−1^ DW, upon exogenously applying a small amount of proline, which appears to amplify the endogenous natural response [63].

In some cases, Pro accumulation in response to salt stress depends on the nature of the toxic ion. For example, in *Tagetes minuta* seedlings grown in the presence of NaCl or KNO_3_, the accumulation of Pro revealed a distinct, ion-specific physiological response to salinity. While both treatments required osmotic adjustment, exposure to NaCl triggered a significantly more aggressive defence, resulting in a ca. 6-fold increase in Pro content, compared to only a 3-fold increase under equimolar KNO_3_ concentrations. This difference suggests that the higher toxicity of NaCl requires a substantially higher concentration of compatible solutes not only to achieve effective osmoregulation but also to ensure cell protection against NaCl-induced damage. Alongside Pro, the authors measured other potential osmolytes, noting significant parallel accumulations of soluble proteins and soluble sugars (specifically glucose, mannose, and xylose-arabinose), which further contribute to osmotic adjustment [64].

Several studies carried out in different plant species also addressed the molecular mechanisms leading to Pro accumulation in response to salt, water deficit or other abiotic stress conditions. These experiments generally showed the stress-induced transcriptional activation of the gene encoding pyrroline-5-carboxylate synthetase (P5CS), the enzyme catalysing the rate-limiting step in the synthesis of Pro from glutamic acid, as well as the inhibition of Pro degradation due to the reduced activity of Pro dehydrogenase (ProDH). The activation of Pro biosynthesis and inhibition of its catabolic pathway has been demonstrated in litchi fruits subjected to chilling stress [65], *Medicago sativa* under salinity stress [66], salt- and drought-stressed *Arabidopsis thaliana* plants [67,68,69], or in *Paoenia ostia* and tomato under severe drought conditions [70,71], to give only a few examples.

The examples mentioned in this section are only a small selection of published reports showing the stress-induced accumulation of Pro to concentrations high enough to assume that they exert a significant osmotic effect, thus supporting the generally accepted notion of Pro as a *bona fide* functional osmolyte. Some additional examples are shown in Table 1.

It should be noted, however, that generally no enough data are provided to calculate the contribution of Pro to total osmotic potential, compared to that of other osmolytes and inorganic ions; this contribution can be relatively modest, even when Pro accumulates to high absolute concentrations. In addition, although we have mainly focused on Pro’s function on osmotic adjustment, lower Pro concentrations do not exclude a significant contribution to stress tolerance mechanisms through its additional roles as an osmoprotectant, ROS scavenger or signalling molecule [22].

## 5. Examples Where Proline Is Not Involved in Osmotic Adjustment Under Stress

Proline accumulation is frequently labelled as a ubiquitous stress response, and quite often published reports highlight the relative stress-induced increase in Pro concentration, rather than its absolute value, to support a functional role of this compound in the stress tolerance mechanisms. However, in many plant species, abiotic stress simply does not trigger a significant increase in Pro contents, or, even if it does, the maximum concentrations reached are still too low to contribute to cellular osmotic balance.

**Figure 5:**
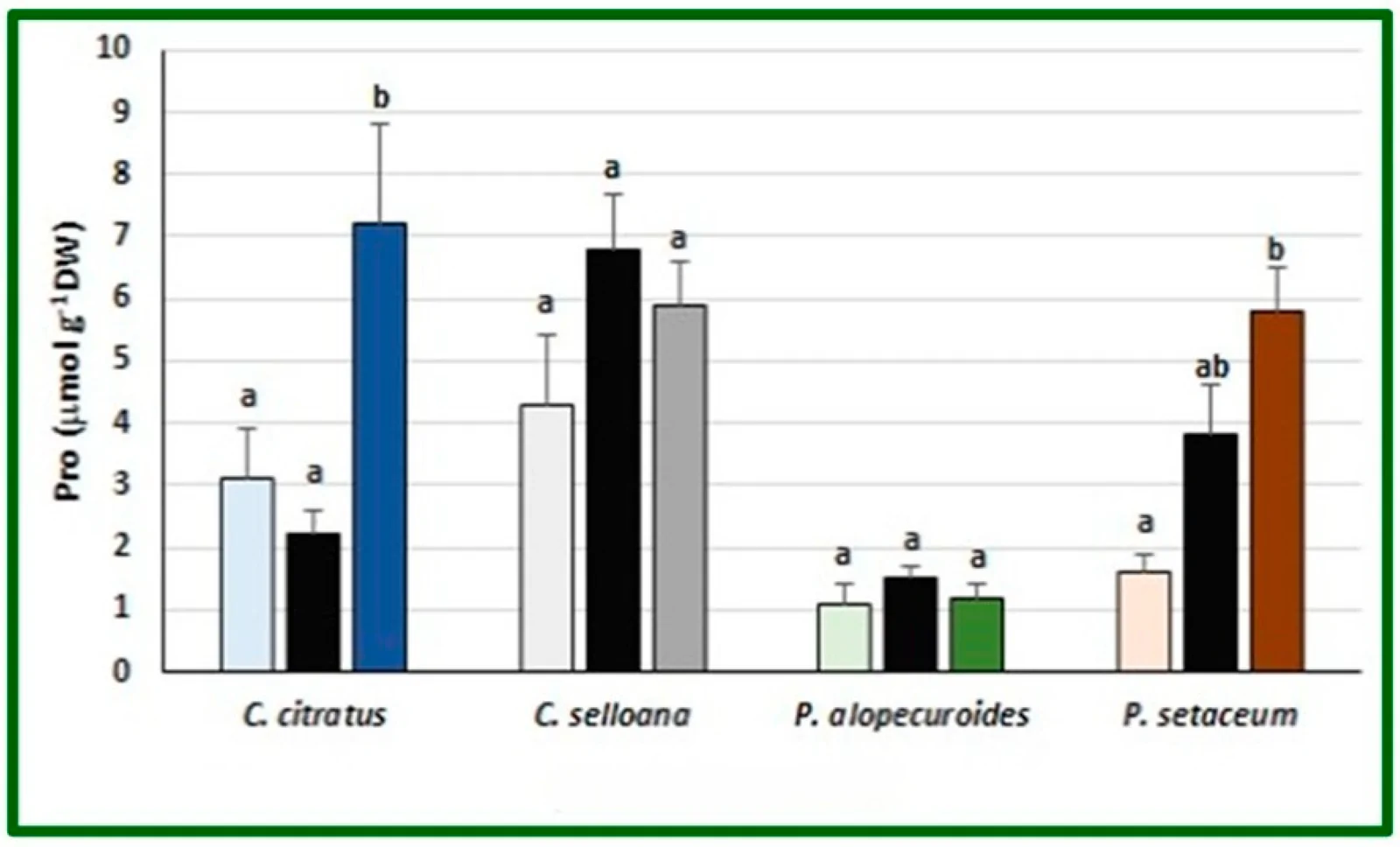
Effect of one-month water stress treatments on the shoot contents of proline (Pro) in *Cymbopogon citratus*, *Cortaderia selloana*, *Pennisetum alopecuroides* and *P. setaceum*. For each species, columns from left to right: 1. control, well-watered plants; 2. intermediate water stress (plants watered with half the volume than control plants); 3. severe water stress (plants not watered at all). Values shown are means ± SE (*n* = 5). Different lowercase letters indicate significant differences between treatments for each species, according to the Tukey test (*p* < 0.05). Adapted from [76].

Some specific plant genera seem to accumulate exclusively other osmolytes, rather than Pro, in response to abiotic stress, such as glycine betaine in *Salicornia* species or sorbitol in *Plantago*. However, in general, there is no correlation between the use of Pro as a functional osmolyte and plant taxonomy, and examples of Pro non-accumulators have been reported across different plant families. For instance, a study analysing the drought stress responses of four potentially invasive ornamental grass species (*Cymbopogon citratus*, *Cortaderia selloana*, *Pennisetum alopecuroides* and *P. setaceum*) [76] showed that water deficit did not trigger any increase in Pro contents in *C. selloana* and *P. alopecuroides*, whereas *C. citratus* and *P. setaceum* exhibited significant increases of 2.3-fold and 3.6-fold, respectively, under severe water stress conditions. However, the absolute Pro concentrations remained remarkably low in all cases (below 8 μmol g^−1^ DW), levels that are insufficient to provide any substantial osmotic effect (Figure 5). Instead, drought tolerance in these species relies heavily on the active transport of Na^+^ and K^+^ cations to the aerial parts, along with increased root K^+^ concentrations in the most tolerant taxa [76].

A similar lack of response has been observed in one-year-old *Abies alba* (silver fir) seedlings, where Pro levels were low (about 15 μmol g^−1^ DW) under control conditions and did not vary significantly after one month of water stress. Salt treatments, up to 300 mM NaCl, induced a modest increase, less than twofold, in Pro contents, not enough to contribute significantly to osmotic adjustment. These results indicate that Pro plays no functional role in the stress responses of this species, which relies instead on the accumulation of high levels of soluble sugars to respond to drought and high salinity stress [77]. Interestingly, as mentioned above for other species, *A. alba* seedlings exhibit relatively high (~400 μmol g^−1^ DW) Na^+^ concentrations in their needles under both control and water-stress conditions, suggesting a constitutive mechanism of stress tolerance. As long as it does not reach toxic levels in the cytosol, Na^+^ can contribute to osmotic balance as a ‘cheap‘ osmoticum since, in terms of energy consumption, ion transport will be less demanding than the de novo synthesis of organic osmolytes [78].

Several Mediterranean halophytes show a strict reliance on alternative osmolytes rather than Pro. Field studies in salt marshes in La Albufera Natural Park, close to the city of Valencia (Spain), on *Sarcocornia fruticosa*, *Inula crithmoides*, and *Plantago crassifolia* [79] revealed that even during peak summer stress, absolute Pro levels remained extremely low (around 2 to 3 µmol g^−1^ DW). Glycine betaine is the primary functional osmolyte in *S. fruticosa* and *I. crithmoides*, reaching concentrations much higher than those of Pro, about 500 and 300 µmol g^−1^ DW, respectively [79]. On the other hand, sorbitol is the major organic osmolyte in the genus *Plantago* and accumulated up to ca. 350 µmol g^−1^ DW in *P. crassifolia* during the most stressful summer season included in the field study [80].

**Figure 6:**
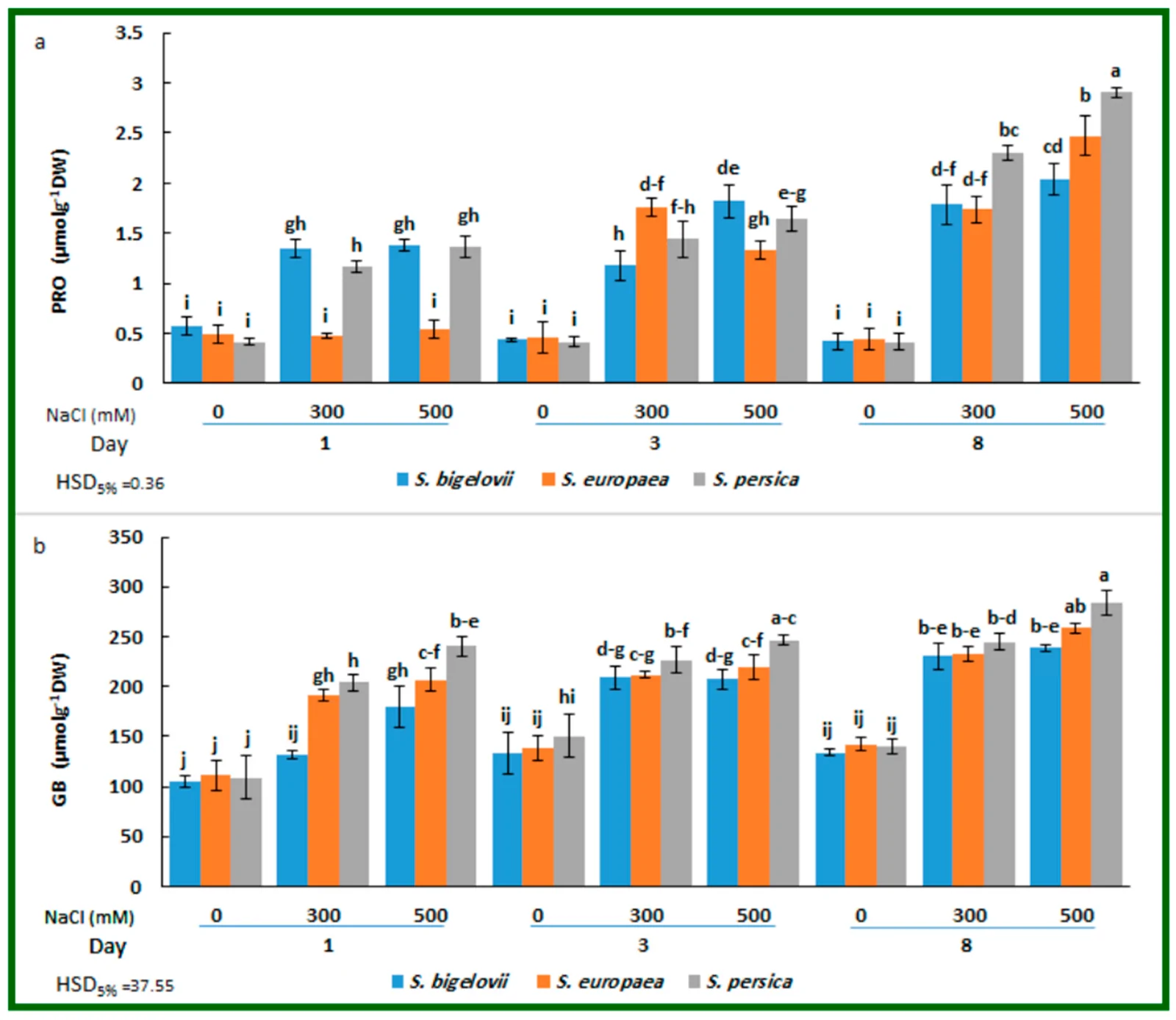
Effects of salt treatments (0, 300, and 500 mM NaCl) on proline (PRO) (**a**) and glycine betaine (GB) (**b**) shoot contents of three *Salicornia* species (*S. bigelovii*, *S. europaea*, and *S. persica*) at three sampling times (1, 3, and 8 days after starting the treatments). The values are means ± SD (*n* = 3). In each panel, different lowercase letters over the bars indicate significant differences between mean values, according to the Tukey test (*p* < 0.05). Taken from [81].

The above data are supported by experiments in which three *Salicornia* species, closely related to *Sarcocornia fruticosa*, were subjected to salt stress under controlled greenhouse conditions [81]. The salt treatments triggered increases in shoot Pro contents in all three species, in a time- and concentration-dependent manner (Figure 6). Under the strongest stress conditions tested (eight days in the presence of 500 mM NaCl), a significant increase in Pro contents was observed, between 4- and 6-fold higher than in the non-stressed controls; however, absolute Pro concentrations reached only 2–3 µmol g^−1^ DW (Figure 6). The applied salt treatments also induced a significant GB accumulation, but with concentrations up to ca. 250 µmol g^−1^ DW; that is, about 100-fold higher than those of Pro. It should be noted that, as indicated before for *Limonium augustebracteatum*, background levels of GB were relatively high, over 100 µmol g^−1^ DW, in the absence of salt, resulting in a salt-induced relative increase of only about two-fold (Figure 6), highlighting that the absolute concentration of the osmolyte, and not its relative increase over control values, is the parameter to be considered when comparing the effects of different compatible solutes on stress responses.

Two *Tamarix* species (*T. aphylla* and *T. jordanis*) provided another example of Pro not exerting a significant osmotic role. A combined salt and drought stress treatment induced 5- to 9-fold increases in Pro concentration over the non-stressed controls. However, the authors calculated that Pro only contributed roughly 0.03 MPa to the cell’s osmotic potential, falling far short of the required 0.2 to 0.4 MPa [82]. Soluble sugars appear to act as the primary osmolytes, whereas Pro serves merely as a secondary osmoprotectant or temporary nitrogen sink. There are many other published studies reporting large relative increases in Pro concentrations in different plant species under varied abiotic stress conditions, supporting the use of this amino acid as a reliable stress biomarker, but without reaching absolute concentrations high enough to contribute substantially to osmoregulation under stress. For example, a ca. 14-fold rise in Pro levels has been reported in peanut (*Arachis hypogaea* L.) plants subjected to a combined salt and drought stress treatment, or a 7-fold increase in response to the salt treatment applied independently, but Pro contents remained below 10–20 µmol g^−1^ DW [83]. Similarly, under hypoxic stress, root Pro contents in rice surged from a baseline of 100–120 µg g^−1^ DW (0.87–1.04 µmol g^−1^ DW) to a maximum peak of 260–310 µg g^−1^ DW (2.26–2.69 µmol g^−1^ DW) by day three, functioning alongside elevated levels of glutamate and total free amino acids. This ABA-mediated Pro accumulation, although irrelevant for osmotic regulation, positively correlated with overall stress resilience in the tolerant ‘Nipponbare’ cultivar, effectively suppressing oxidative damage markers (MDA and carbonyl groups) while selectively upregulating specific antioxidant enzyme activities like POD and APX [41]. Some additional reports showing that Pro is not involved in cellular osmoregulation under stress are summarised in Table 2.

## 6. Comparative Studies in Genetically/Taxonomically Related Taxa and Correlation with Their Relative Tolerance

For those—many—plant species for which application of a particular stress results in a significant increase in Pro content, there is generally a positive correlation between the intensity of the stress and the concentration of accumulated Pro, supporting its use as an abiotic stress biomarker. However, this does not mean that Pro is directly involved in the mechanisms of tolerance, even though this is often assumed in the literature. On the other hand, evaluating Pro accumulation in response to specific stress treatments across genetically or taxonomically related taxa showing different degrees of tolerance represents a useful approach to assess the possible functional role of Pro in those tolerance mechanisms. When comparing the responses of several related genotypes, such as different populations, cultivars or subspecies of a particular species, or different species of the same genus, correlation of Pro contents with their relative degree of tolerance may reveal a profound divergence in Pro functional role. The correlation could be positive, with Pro reaching higher levels in the most tolerant genotypes, supporting its direct involvement in tolerance mechanisms. On the contrary, Pro concentration could be relatively higher in the most sensitive genotypes, which would be more stressed than tolerant ones under the same conditions; in this case, Pro is simply a stress biomarker but probably does not contribute substantially to tolerance. In addition, there are unclear cases where no straightforward correlations between Pro accumulation levels and the relative degree of stress tolerance could be established.

### 6.1. Positive Correlations: High Pro Accumulation as a Determinant of Tolerance

In several plant genera, Pro accumulation in response to stress can be used to directly distinguish stress-tolerant species from their sensitive relatives. Comparative studies on *Juncus* species effectively revealed a positive correlation between Pro accumulation and stress tolerance, distinguishing two halophytes, *J. maritimus* and *J. acutus*, growing in saline and arid natural environments, from *J. articulatus,* a species adapted to non-saline, humid habitats. Under high salinity conditions (400 mM NaCl), Pro content in the halophytes surged dramatically, increasing between 60- and 70-fold compared to non-stressed controls, reaching concentrations of ca. 120 µmol g^−1^ DW after eight weeks of treatment. In contrast, the sensitive *J. articulatus* exhibited a weak response, with maximum Pro levels remaining below 10 µmol g^−1^ DW under the same conditions [16]. A similar pattern was observed under water stress (eight weeks without irrigation), where the halophytes again demonstrated a robust response, reaching Pro levels over 100, or close to 200 µmol g^−1^ DW, in *J. acutus* and *J. maritimus*, respectively, whereas Pro contents below 10 µmol g^−1^ DW were measured again in *J. articulatus.* Pro is not the only osmolyte contributing to counteract the osmotic stress caused by high salinity or water deficit in *Juncus* species. Sucrose levels also increase significantly in response to both treatments, but without the stark differences observed for Pro as it accumulated similarly across the three species, with maximum concentrations between 150 and 250 µmol g^−1^ DW. These results strongly support Pro accumulation as a key mechanism of stress tolerance in *Juncus* [16].

**Figure 7:**
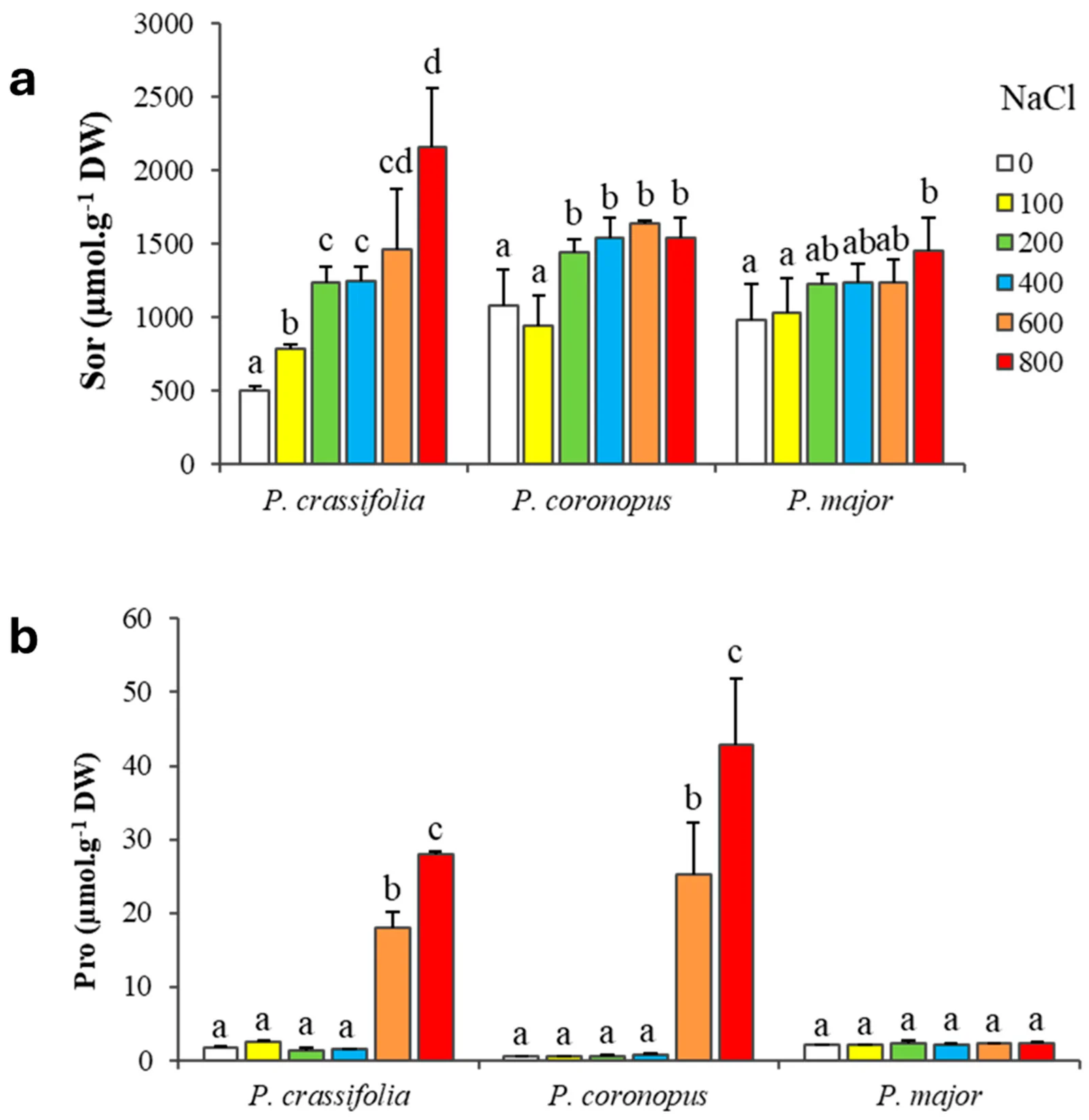
Sorbitol (Sor) (**a**) and proline (Pro) (**b**) leaf contents after four weeks of treatment with the indicated NaCl concentrations in the three selected *Plantago* species (means ± SD, *n* = 5). Different lowercase letters indicate significant differences between treatments for each species, according to Tukey test (α = 0.05). Taken from [93].

Similar responses to salt stress have been reported when comparing two salt-tolerant members of the genus *Plantago*, *P. crassifolia* and *P. coronopus*, with the congeneric glycophyte *P. major* [93]. As mentioned before, sorbitol is the identified functional osmolyte in this genus. Background sorbitol levels in the absence of stress are already quite high and increase with the increase of NaCl concentration in irrigation water, but to a similar extent in the three analysed species, reaching maximum values between 1500 and 2000 µmol g^−1^ DW (Figure 7a). Therefore, although sorbitol is clearly the major compatible solute responsible for osmotic adjustment under stress, its accumulation patterns cannot explain the differences in salt tolerance. Pro accumulation, on the other hand, is a distinctive response to high salinity triggered specifically in the halophytes. As determined in the field [79], background Pro levels under non-stress conditions are extremely low, below 2 µmol g^−1^ DW, a concentration that does not increase upon controlled salt treatments up to 400 mM NaCl. However, Pro levels rise significantly at higher salinities, but only in the halophytes, up to 30 µmol g^−1^ DW in *P. crassifolia* and to 45 µmol g^−1^ DW, approximately, in *P. coronopus* (Figure 7b) [93]. The positive correlation between Pro content and the relative salt tolerance of these species suggests its direct participation in the mechanisms of tolerance. Since the contribution of Pro to osmotic balance is rather modest, compared to that of sorbitol, Pro function must be mostly based on its additional roles as an osmoprotectant, ROS scavenger and signalling molecule. It should also be noted that the salt concentrations that trigger Pro accumulation in the halophytes in the controlled treatments are higher than those that the plants must normally withstand in their natural habitats. In this genus, Pro biosynthesis can be considered as a “security switch”, which would be activated under natural conditions only as a response to an extreme, probably temporary increase in soil salinity, which is not unlikely in the current climate change scenario.

This positive correlation between Pro accumulation under stress and the relative tolerance of different related genotypes extends to cultivars of agricultural crops. For example, the exposure to 60 mM NaCl of different potato (*Solanum tuberosum*) cultivars induced Pro accumulation in leaf and stem tissues, with the highest concentrations localised in the stem. Notably, the salt-tolerant cultivar Desiree accumulated approximately three times more Pro in its stem than the sensitive cultivar Mozart, due to a twofold increase in the expression of the biosynthetic gene *P5CS1*, a response lacking in Mozart [94]. In Tunisian tomato (*Solanum lycopersicum*) genotypes subjected to a seven-day salt treatment, the salt-tolerant cultivar San Miguel showed higher Pro contents in roots and leaves than the salt-sensitive cultivar Mouna HF1, so that Pro accumulation was considered a suitable marker for salt tolerance that could be applied in breeding programmes [95]. There are many more published reports, including some classical articles, comparing Pro levels in cultivars of major crops differing in their tolerance to stress, mostly in response to drought, and showing similar positive correlations. For example, in barley [96,97], spring wheat [98], or maize [99,100].

### 6.2. Negative Correlations: Pro Accumulation as a Symptom of Stress Severity

A substantial subset of comparative literature reveals that robust Pro synthesis frequently correlates negatively with stress resilience, opposite to the previous examples. In these contexts, massive Pro accumulation is not a proactive defence mechanism but rather a symptom of cellular damage due to high stress sensitivity.

This negative correlation is documented, for example, by several studies on *Phaseolus*. Three cultivars of common bean (*P. vulgaris*) and one of runner bean (*P. coccineus*) were subjected to increasing salt concentrations, up to 150 mM NaCl, for three weeks. Determination of growth parameters allowed the establishment that the common bean cultivar Maxidor was the most salt-tolerant, followed by the runner bean Moonlight, whereas The Prince was the most sensitive. Leaf Pro contents were low and similar in non-stressed plants of all cultivars and increased in the salt-treated plants up to >40 µmol g^−1^ DW in The Prince, but only to ca. 10 µmol g^−1^ DW in Maxidor. These results suggested that Pro is not directly involved in the mechanisms of salt tolerance in these cultivars, which depend instead on blocking Na^+^ (and Cl^−^, to a lesser extent) transport from roots to shoots, *myo*-inositol accumulation in leaves and, at high external salinities, activation of K^+^ transport to the leaves [101]. A similar pattern was maintained under water stress conditions, where the most tolerant cultivar, again Maxidor, showed the lowest Pro accumulation [102].

**Figure 8:**
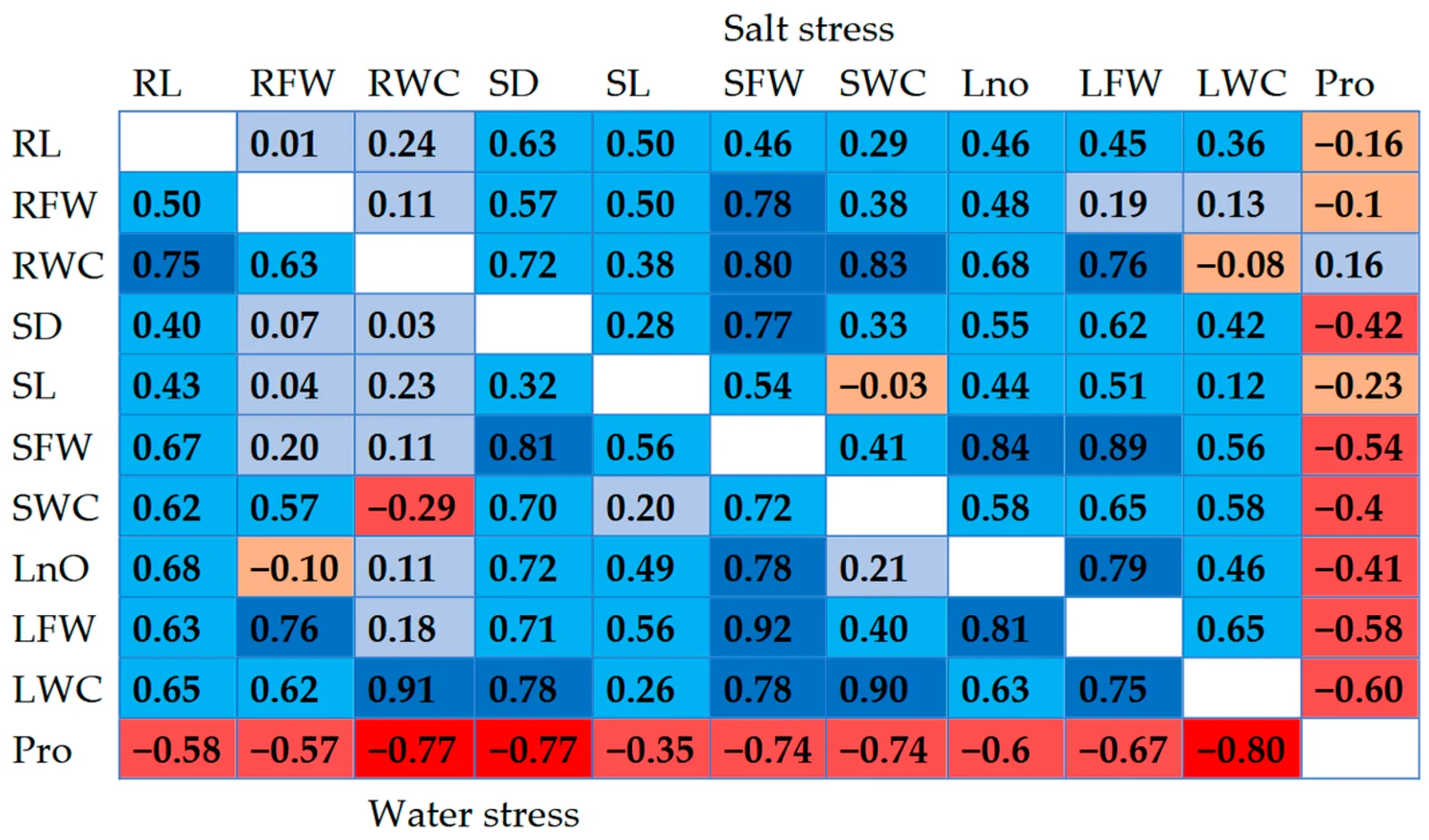
Heatmap of Pearson moment correlation coefficient (r) between the analysed traits in *Phaseolus vulgaris* genotypes submitted to two weeks of water (no irrigation) and salt (150 mM NaCl) stress treatments. Dark blue denotes high positive correlation, and dark red high negative correlation. Abbreviations: RL, root length; RFW, root fresh weight; RWC, root water content; SD, stem diameter; SL, stem length; SFW, stem fresh weight; SWC, stem water content; Lno, leaf number; LFW, leaf fresh weight; LWC, leaf water content; Pro, proline content. Adapted from [10].

The above results prompted a wider analysis of 47 *P. vulgaris* genotypes, including local landraces, commercial cultivars, and experimental lines, from Spain (23), Colombia (19), and Cuba (5), which were subjected to two or three weeks of salt stress (150 mM NaCl) or water stress (complete withholding of irrigation) treatments. For all genotypes, leaf Pro contents increased significantly in response to both stresses. Pro showed strong negative correlations with all determined growth parameters, especially with leaf/stem fresh weight and water content, as confirmed by PCA loading plots, with higher Pro concentrations unequivocally linked to stronger growth inhibition and stress sensitivity. Figure 8 shows the heatmap of Pearson correlation coefficients for the two-week stress treatments. These results support the use of Pro content determination as a simple, rapid and reliable marker for large-scale screening to exclude sensitive cultivars in breeding programmes for drought and salt tolerance in *Phaseolus* [10].

Similar negative correlations have been observed in the analysis of nine accessions of “Ardhaoui” barley landraces from different regions of Tunisia, subjected to water stress by completely stopping irrigation for three weeks. Large increases in Pro levels were measured in all accessions, although with wide quantitative differences, as Pro concentrations ranged from nearly 150 to about 600 μmol g^−1^ DW. These high values indicated that Pro must contribute to osmoregulation under drought, together with soluble sugars, which also increased in the water-stressed plants. However, the most tolerant accession 2, from El May Island, exhibited the lowest Pro levels, whereas the highly susceptible accession 4, from Ksar Hdada mountain, showed the highest levels [12]. This study casts doubt on the utility of Pro as a reliable general marker of drought tolerance in barley genotypes, suggested by previous reports showing opposite correlations [96,97], and the authors proposed that the reduction of chlorophylls or MDA contents, or the increase in total phenolic compounds, could be used as more appropriate parameters for selecting drought-tolerant barley varieties [12]. On the other hand, this work supports previous studies with different barley genotypes subjected to salt stress, which also showed similar negative correlations, suggesting that hyperaccumulation of Pro or other major compatible solutes in barley does not play a major role in salt-tolerance but is rather a symptom of salt susceptibility [74]. In any case, these data highlight the risk of generalising results obtained independently, even with different varieties or cultivars of the same species.

In another study, drought responses were analysed in several commercial cultivars of three *Tagetes* (marigold) species, *T. patula*, *T. tenuifolia* and *T. erecta*, by subjecting the plants to three weeks without irrigation. The stress treatment induced a significant increase in Pro contents, in all genotypes, with quantitative differences observed between species and between cultivars within each species. For most cultivars, Pro levels in stressed plants increased between 30 and 70-fold compared to the corresponding controls, reaching concentrations ranging from ca. 50 to 350 μmol g^−1^ DW. Determination of additional variables and Principal Component Analysis allowed identification of the most tolerant and sensitive cultivars within each species, and it could be concluded that Pro was negatively correlated with the relative degree of tolerance: higher levels of Pro were detected in the more sensitive genotypes of the same species—for example, *T. erecta*’s “Cupid Golden Yellow”, or *T. tenuifolia*’s “Luna Orange” and “Luna Lemon” [14].

In the above examples, the participation of Pro in stress tolerance mechanisms cannot be ruled out, either by contributing with other osmolytes to osmoregulation, as a ROS scavenger or as a signalling molecule. However, other stress responses, different from Pro accumulation, must be responsible for the observed differences in tolerance between closely related genotypes.

## 7. Summary and Perspectives

Proline contents generally increase in parallel to the intensity of the applied stress treatment, supporting its use as a general stress biomarker, even though the maximum concentrations reached can be relatively low. However, its accumulation in response to stress conditions, such as water deficit, salt treatments, chilling or too high temperatures, does not necessarily imply a direct role in stress tolerance mechanisms. Because these responses are often shaped by species-specific genetics and adaptations to particular environments, broad generalisations are inappropriate, and the functional significance of Pro should be evaluated on a case-by-case basis.

To avoid a “Pro-centric” perspective, researchers should assess possible stress-induced changes in the concentration of a broader spectrum of osmolytes—including glycine betaine, polyalcohols, and soluble sugars—rather than focusing exclusively on Pro. This problem has been addressed in recent works through metabolomics approaches (e.g., [103]), but the major limitation—i.e., the quantification of these metabolites in subcellular compartments—remains challenging as quantitative subcellular metabolomics is still in its infancy, particularly for small and highly dynamic metabolites such as proline [104].

Another key flaw in Pro literature is that static measurements are generally presented, treating pool size as a proxy for function and disregarding the dynamic nature of Pro metabolism, which may be critical for stress tolerance. Recent developments in isotopically non-stationary metabolic flux analysis have made it possible to quantify intracellular metabolic fluxes in plant systems [105] and could be applied in future work to Pro metabolism, helping clarify its physiological role in abiotic stress responses.

Apart from quantifying organic osmolytes, inorganic ion contents should also be determined, as they are, in general, the main contributors to total cellular osmotic potential. As shown by some of the examples included in this review, inorganic ions can accumulate at relatively high concentrations, either constitutively or in response to different stress treatments, but they are generally not measured—except, obviously, when assessing plant responses to salt stress.

Equally important, future studies should standardise osmolyte reporting (and other variables) to molar concentrations on a dry weight basis (e.g., µmol g^−1^ DW) to ensure physiological relevance and facilitate meaningful comparisons across experiments. Without dry weight standardisation, changes in tissue hydration under stress can artificially distort fresh weight–based metabolite measurements, thereby reinforcing the very misinterpretations highlighted in this review.

Finally, whenever feasible, studies should incorporate comparative analyses of related taxa that differ in stress resilience. Linking relative tolerance levels with absolute Pro concentrations is essential for determining whether Pro functions as an active adaptive defence mechanism or simply reflects physiological stress and damage.

In conclusion, the general use of proline as a reliable biochemical marker of abiotic stress in plants is widely supported in the literature. However, its contribution to stress responses and plant survival, and the mechanisms involved, are strongly context-dependent and cannot be generalised across different species.

## Figures and Tables

**Table 1:** Proline accumulation in various plant species in response to abiotic stress treatment and additional stress-induced changes in other stress biomarkers. Abbreviations for other osmolytes: GB (glycine betaine); TSS (total soluble sugars); for oxidative stress biomarkers: MDA (malondialdehyde); for antioxidant metabolites: TPC (total phenolic content), TF (total flavonoids). Symbols: ↑ = increased; ⇿ = no significant change.

Species	Stress Treatment	Pro Content in Control (μmol g^−1^ DW)	Maximum Pro Content Under Stress (μmol g^−1^ DW)	Additional Stress-Induced Changes	Ref.
*Hordeum vulgare*	Drought	150	600	TSS ↑, MDA ↑, TPC ↑, TF ⇿	[12]
*Tagetes patula*	Drought	5.34	243.65	GB, TSS ↑ MDA ↑, TPC ↑, TF ↑	[14]
*T. erecta*		9.36	152.15		
*T. tenuifolia*		2.52	229.52		
*Raphanus sativus* cv. National	Drought	21.90	224.10	-	[72]
*Solanum lycopersicum* cv. Dafnis	Salt stress	ca. 13 *	174–217 *	-	[73]
*S. lycopersicum* cv. Maxifort					
*S. lycopersicum* cv. BKO					
*S. lycopersicum* cv. B-blocking					
*Hordeum vulgare* cv. Numar	Salt stress	ca. 10	ca. 300	GB ↑	[74]
*H. vulgare* cv. ZUG293			ca. 100		
*H. vulgare* cv. Gaimder			ca. 450		
*H. vulgare* cv. ZUG403			ca. 400		
*Capsicum annuum* L.	Heat stress	ca. 10	ca. 90	-	[75]

(*) Pro concentrations are expressed in mg g^−1^ DW in the original publication and converted to µmol g^−1^ DW for standardisation.

**Table 2:** Some examples of proline accumulation under stress at concentrations too low to have a significant osmotic effect and additional stress-induced changes in other stress biomarkers. Abbreviations for other osmolytes: GB (glycine betaine); TSS (total soluble sugars); for oxidative stress biomarkers: MDA (malondialdehyde), H_2_O_2_ (oxygen peroxide); for antioxidant metabolites: TPC (total phenolic content), TF (total flavonoids); antioxidant enzymes: SOD (superoxide dismutase), CAT (catalase), POD (peroxidase), APX (ascorbate peroxidase), GR (glutathione reductase). Symbols: ↑ = increased; ↓ = decreased; ⇿ = no significant change.

Species	Stress Treatment	Pro Content in Control (μmol g^−1^ DW)	Maximum Pro Content Under Stress (μmol g^−1^ DW)	Additional Stress-Induced Changes	Ref.
*Sesamum indicum*	Drought	2.64	4.51	TSS ⇿; GB ↑, MDA ⇿, H_2_O_2_ ↑	[84]
*Oryza sativa* cv. Cakrabuana	Drought	16.8	24.8	-	[85]
*O. sativa* cv. Ciherang		14.1	21.2		
*O. sativa* cv. IR64		12.5	18.7		
Hot pepper cv. NW Bigarim	Heat stress	1.7 *	3.9 *	-	[86]
Hot pepper cv. Chyung Yang		2.4 *	2.4 *		
*Capsicum annuum* cv. Geumsugangsan	Salt stress	23.80 *	61.67 *	TSS ↓, ↑, H_2_O_2_ ↑, SOD ↑, POD ↑, TPC ↑, TFC ↑	[62]
	Drought		28.84 *		
	Cd stress		31.88 *		
*Silene vulgaris*	Salt stress	2–10	ca. 10	TSS ↑, MDA ⇿, TPC ↑, TF ⇿ (↓, under drought stress)	[87]
	Drought		ca. 2		
*S. sclerocarpa*	Salt stress		ca. 30		
	Drought		ca. 15		
*S. latifolia*	Salt stress		ca. 30		
	Drought		ca. 40		
*S. gallica*	Salt stress		ca. 50		
	Drought		ca. 10		
*Prunus* rootstocks	Drought	4.34 *	10.42 *	TSS ⇿	[88]
*Solanum lycopersicum*	Salt stress	-	ca. 60	-	[89]
*S. lycopersicum* cv. Kosaco	Drought	17.37 *	21.71 *	-	[90]
*S. lycopersicum* cv. Josefina		13.03 *	17.37 *		
*S. lycopersicum* cv. Katalina		17.37 *	26.06 *		
*S. lycopersicum* cv. Salome		13.03 *	17.37 *		
*S. lycopersicum* cv. Zarina		13.03 *	13.03 *		
*Cicer arietinum*	Drought	0.26	1.04	CAT ↑, SOD ↑, POD ↑, APX ↑, GR ↑	[91]
*Triticum durum*	Salt stress	0.08	18.8	-	[92]
	Drought	0.93	48.66		

(*) Proline concentrations were expressed in µg g^−1^ DW or mg g^−1^ DW in the original publications and converted to µmol g^−1^ DW for standardisation.

## Data Availability

No new data were created or analyzed in this study. Data sharing is not applicable.
